# Highlights from the 2015 WIN Symposium: novel targets, innovative agents, and advanced technologies—a WINning strategy?

**DOI:** 10.3332/ecancer.2015.564

**Published:** 2015-08-13

**Authors:** Richard L Schilsky

**Affiliations:** American Society of Clinical Oncology, Alexandria, Virginia 22314, USA

**Keywords:** biomarkers, genomics, personalised medicine, proteomics, targeted therapy

## Abstract

The worldwide innovative networking (WIN) consortium comprises a global alliance of 28 academic and clinical cancer centres, 11 pharmaceutical and technology companies and five charitable or health payer organisations. Since its inception the consortium has striven to provide a forum for all of its members to network, share information and experience, and perform clinical trials with the overarching goal of advancing the care of patients with cancer through the use of precision medicine.

The annual 2-day WIN Symposium is the most visible output of the consortium and provides an opportunity for around 400 experts and other delegates to meet and discuss the latest research and initiatives in personalised cancer medicine. The seventh WIN Symposium, held in Paris, France, 29–30 June 2015, consisted of nine plenary and eight poster sessions that covered the overarching theme of novel targets, innovative agents, and advanced technologies being a winning strategy.

Highlights included discussions of immune mechanisms and ways to target the cancer immunome and systems biology approaches to supporting personalised cancer. The latest data from the BATTLE-2 and WINther trials were discussed, and round table discussions were held that focused on how best to design the next generation of clinical trials, which included SPRING, SUMMER, and BOOSTER being initiated by the WIN Consortium.

## The era of precision medicine: a worldwide effort and challenge

During the first plenary session, the Chairman of the WIN Consortium John Mendelsohn (The University of Texas MD Anderson Cancer Centre, Houston, TX, USA) provided an overview of the WIN Consortium and the global effort needed to improve patient access to precision medicine. The premise behind the creation of the Consortium was that this is too big a task for one institution alone and that a worldwide network assembling all cancer stakeholders to develop cutting-edge concepts in precision cancer medicine was needed to have a real impact on patient survival. Since its inception in 2010, WIN has worked to share information, promote investigation, and expand clinical adoption of personalised cancer therapy via the annual WIN Symposium and publications. The launch of the WINther trial in several European countries and the planned SPRING, SUMMER, and BOOSTER trials are a testament to the successful collaboration that it has been possible to achieve to date.

## Targeting immune mechanisms

We are only just beginning to realise the promise of immune therapies in treating cancer and the fact that some immune therapies seem to be effective against essentially every kind of cancer irrespective of histological type or mutational status. The focus of the second plenary session was approaches being undertaken to identify and understand immune mechanisms and try to understand how these may be harnessed to improve upon the successes seen with immunotherapies to date. James Allison (The University of Texas MD Anderson Cancer Centre, Houston, TX, USA) highlighted in a keynote lecture that the only thing perhaps more complicated than cancer is the immune system itself. His talk touched upon the ability of cancer to generate heterogeneous subclones that can easily be matched by the ability of the immune system to generate enormously heterogeneous immune responses.

Because of the great expense associated with modern cancer therapies it is becoming more important than ever before to be able to identify the patients most likely to benefit from any particular type of treatment. Angel Porgador (Ben-Gurion University of the Negev, Beer Sheva, Israel) presented some of his research looking at the cancer immunome and how this may be used to identify signatures of immune response. Ton Schumacher (The Netherlands Cancer Institute, Amsterdam, The Netherlands) then discussed data providing a strong incentive for the development of a new generation of personalised immunotherapies that exploit cancer genome information to target patient-specific mutant antigens.

## Systems biology and new approaches to support personalised cancer care

Andrea Califano (Columbia University, Department of Systems Biology, New York, USA) gave a keynote lecture to start off the third plenary session, looking at using a systems biology approach to precision cancer medicine.

Dr Califano’s lecture highlighted the enormous complexity of cancer and the fact that it may be possible, through very careful and innovative analysis of molecular networks, to identify key nodal points where there are master regulators of those regulatory pathways that, if targeted, could potentially have a major impact on the treatment of cancer.

Personalised, genomic-guided combination therapy and its potential to transform disease management in ovarian and breast cancer was the subject of a presentation given by Brian Leyland-Jones (Avera Cancer Institute, Sioux-Falls, USA). The strategy involves multiplatform technology and the use of specific algorithms to decide which combinations of drugs would best suit a particular patient. Dr Leyland-Jones explained that the aim is to hopefully show regulatory agencies, health authorities and medical insurance companies that the addition of genomic-guided therapy to standard therapy in early-stage disease can transform the outcome of therapy.

Also, during this session, Waun Ki Hong (The University of Texas MD Anderson Cancer Center, Houston, TX, USA) gave an overview of the biomarker-integrated approaches of targeted therapy for lung cancer elimination (BATTLE) 2 trial. The first BATTLE trial set a new standard for personalised cancer medicine trials in that it was the first prospective trial to require core biopsies and subsequent biomarker analysis to select treatment [1]. The trial was a bold step towards improving the efficiency of biomarker-based drug development, Dr Hong observed, and served to galvanise the whole field of precision medicine and influence the design of subsequent trials, including the BATTLE-2 trial.

## Next-generation trials with targeted therapies

Strategies to help overcome drug resistance and how best to plan future trials was the focus of an academic and industry expert round table discussion held during the fourth plenary session. Joining Dr Mendelsohn, Dr Hong, and myself on the panel were Alexander Eggermont (Cancer Institute Gustave Roussy, Villejuif, France) who is Vice-Chair of the WIN Consortium, Razelle Kurzrock (University of California, San Diego, USA) who is the Chair of the WIN Consortium Clinical Trials Committee, Jean-Charles Soria who is the Chair of Drug Development Department at Cancer Institute Gustave Roussy and PI of the WINTher trial, and industry representatives Richard E. Buller (Pfizer Oncology, La Jolla, USA), Katherine Galvin (Takeda Oncology, Cambridge, USA) and Antoine Yver (AstraZeneca, Wilmington, USA).

The discussion highlighted some of the challenges faced when combining drugs to try to overcome resistance, such as deciding which drugs to combine, how to develop optimal dosing strategies, consideration of what the combined effect, and toxicities, of the selected agents may be, whether the drugs need to be given concurrently, sequentially and in what order and how to identify potential biomarkers that may be able to help select patients that may benefit from a particular combination.

One of the novel things about the WIN Consortium is that it has been one of the few clinical trial organisations to begin to combine drugs very early in very difficult to treat cancers and approach it in a very innovative way. This is evidenced by the first trial in the Consortium’s portfolio, WINther (A Study to Select Rational Therapeutics Based on the Analysis of Matched Tumour and Normal Biopsies in Subjects With Advanced Malignancies). Interim results and some of the challenges faced [2] and lessons learned from the trial were presented by Professor Soria.

## Beyond WINTHER—future WIN Consortium trials

The fifth plenary session took a forward look at new trials being planned by the WIN Consortium which are going to be conducted in patients with non-small-cell lung cancer (NSCLC), moving from early (Stage I) disease in BOOSTER to the adjuvant setting in SUMMER and the metastatic setting in SPRING.

BOOSTER, which was presented by Harvey Pass (New York University Langone Medical Centre, New York, USA), is a research platform being designed to help with the discovery and validation of new blood biomarkers for the diagnosis of Stage I NSCLC. The overall aim is to try to improve the early diagnosis of the disease. Pre-, peri- and postsurgical biospecimens will be obtained to try to capture the long-term clinical outcomes of these patients. BOOSTER should provide us with a rich data set that can be used to identify those biomarkers that might actually be useful in either identifying NSCLC very early or for monitoring its recurrence.

SUMMER (Elimination of relapse through innovative adjuvant targeted therapies in Stages II and III NSCLC) trial, which was discussed by Don L. Gibbons (The University of Texas MD Anderson Cancer Centre, Houston, USA) is essentially an adjuvant, post-operative therapy trial for patients who have resectable disease and are randomised to the usual standard of care which is chemotherapy versus chemotherapy with one or more targeted agents with the targeted agents to be selected based on biomarkers. The trial’s design, which is currently being finalised, is novel in that it will look at precision medicine and targeted therapies in the adjuvant rather than metastatic setting and it has one study arm that will specifically look at the benefit of using combinations of immunotherapy and targeted agents in patients with NSCLC.

Then, there is SPRING, which Dr Kurzrock (University of California, San Diego, USA) discussed. The ultimate goal of the SPRING trial is to improve the 5-year survival of patients with metastatic NSCLC by giving three targeted drugs in combination. The challenge here is in deciding which three drugs to use, as the three selected drugs may well be different according to individual patient and tumour characteristics. This is where the SIMS (simplified interventional mapping system) algorithm [3] should provide the information needed help guide therapeutic decisions in this and in the other planned suite of next generation WIN trials. SIMS, which was discussed by Vladimir Lazar (Cancer Institute Gustave Roussy, Villejuif, France) at the Symposium, was developed by using targeted genomic sequencing, copy number variation, transcriptomics, and miRNA expression analysis of matched lung tumour and normal lung tissue samples. An algorithm was developed to create a scoring system that allows potential drug targets to be ranked for each patient based on their contribution to pathway activation.

## New avenues: big trials and big data

During the sixth plenary session, James Doroshow (National Cancer Institute, Bethesda, USA) gave a perspective on the National Cancer Institute’s strategy and trials and I had the opportunity to highlight some of ASCO’s recent initiatives in advancing personalised medicine. ASCO’s initiatives broadly fall into two categories: educational programmes and opportunities to learn from real world clinical practice.

With respect to educational programmes, ASCO’s clinical guidelines have increasingly started to focus on the importance and appropriate use of biomarkers in the molecular work-up of newly diagnosed patients with cancer. And, in collaboration with the College of American Pathologists (CAP) and the Association for Molecular Pathology, ASCO recently launched the Molecular Oncology Tumour Board series (http://university.asco.org/motb), which is an online and user-driven resource designed to help clinicians understand and interpret molecular profiling tests and studies. Each month, a new case-based discussion is posted online at ASCO University®, and then, there is a 2-week period where people can comment before an expert analysis by a medical oncologist and clinical pathologist is published.

With the goal of learning from real-word clinical practice, ASCO has recently announced plans to launch the Targeted Agent and Profiling Utilisation Registry (TAPUR) study. This is a clinical study in which patients with advanced solid tumours, multiple myeloma or B-cell non-Hodgkin lymphoma who have undergone genomic profiling that suggests off-label treatment with a commercially available targeted treatment might be of benefit are being studied. The aims of TAPUR are to not only describe the off-label use of available targeted cancer therapies, but also to facilitate patients’ access to these treatments. Then, on a wider scale, ASCO has begun development of CancerLinQ™ (http://cancerlinq.org/) that has the goal of collating the electronic medical records of all patients diagnosed with cancer in the United States. The aggregated information can hopefully be used to learn from real-life clinical practices and be used to understand outcomes of special populations like the elderly, patients with specific genomic types, patients under age 30, or those in poor general health.

Results of a meta-analysis of all available Phase 2 and Phase 3 personalised medicine trials involving 70,000 patients was presented by Dr Kurzrock. Results showed that the use of targeted therapies was as effective, and in some cases more effective, than standard therapeutic approaches in appropriately selected patients. Here, the key was patient selection, as using targeted treatments on an all-comer basis was not just ineffective, it was potentially harmful.

## Multi-modality and multi-drug cancer therapy

Several approaches to improving cancer therapy by using multiple agents or modalities of treatment were discussed during the meeting. One of the most promising perhaps is the idea or combining radiation and immunotherapy. Ralph Weichselbaum (University of Chicago, Illinois, USA) discussed the new therapeutic partnership being forged between the two cancer treatment modalities and how radiation can be used to elicit a systemic immune response that, when combined with immunotherapies such as checkpoint inhibitors, may be harnessed to not only improve the control of local tumour growth, but also potentially address the problem of metastatic disease.

## Recent progress in specific tumour types

Plenary 8 looked at some of the recent progress that has been made in specific tumour types, with Iman Osman (New York University Langone Medical Center, New York, USA) discussing what is new in melanoma. Bin Tean Teh (Duke-NUS Graduate Medical School, Singapore) discussed the discovery of the molecular fingerprint of a known carcinogen aristocholic acid found in certain herbal medicines that is linked to the development of urothelial cancer. Dr Teh also highlighted the discovery of recurrent mutations in exon 2 in the *MED12* gene in breast fibroadenomas, a common benign breast tumour in women.

## Regulatory and funding challenges

The final plenary session before the close of the meeting looked at some of the regulatory and funding challenges when conducting global clinical trials of precision cancer therapies. When conducting trials in multiple countries with various regulatory systems and laws, the challenge becomes finding that common ground that meets all the necessary stipulations to enable a trial to launch in all the individual countries.

One of the issues with the WINther trial, which has still not launched in the United States but has been up and running for several years in Europe, was that the analytical methodology used did not conform to regulatory requirements, as Stanley Hamilton (The University of Texas MD Anderson Cancer Centre, Houston, USA) explained. He highlighted the importance of obtaining appropriate methodology certification and accreditation to perform assays in all countries that plan to participate in a global trial.

One of the key elements of WIN is the collaboration between academia, industry, and regulatory and charitable bodies. This year’s Symposium included talks from Francesco Pignatti (European Medicines Agency, London, UK) on the European Medicines Agency’s adaptive pathways approach to improve timely access for patients to new medicines and from Tatiana Prowell (Centre for Drug Evaluation and Research, US Food & Drug Administration, Bethesda, USA) on end points and assessment of therapeutic efficacy in personalised cancer care.

Carole Redding Flamm (Blue Cross Blue Shield Association, Chicago, USA) spoke about the use of targeted therapies and challenges for health payers and Jacques Raynaud, of the charitable organisation Fondation ARC pour la recherche sur le cancer, Paris, France, spoke about the role of foundations and cancer research organisations in supporting research in the era of precision cancer medicine.

## Poster session highlights

Winner of the best poster awards at this year’s meeting were Sonia Vivas and colleagues (Molecular Health GmbH, Heidelberg, Germany) for their work on determining the prevalence of secondary genetic modifiers on cancer drug biomarkers and implication for the clinical utility of gene-based diagnostics and Marie Dutreix (Institut Curie, Orsay, France) who presented the results of a first-in-human Phase 1 study of the DNA repair inhibitor DT01 in combination with radiotherapy in patients with skin metastases from melanoma.

Other poster highlights included the use of using TargomiRs in the treatment of cancer, with positive results seen in a Phase 1 trial of patients with malignant mesothelioma presented by Nico van Zandwijk and colleagues (University of Sydney, Concord, Australia).

Finally, Jessica Menis (EORTC Headquarters, Brussels) and associates presented a poster on SPECTAlung (http://spectalung.eortc.org), which is a joint initiative of the European Organisation for Research and Treatment of Cancer in collaboration with the European Thoracic Oncology Platform (http://www.etop-eu.org/) that aims to improve clinical trial access for patients with thoracic tumours. The project involves 18 screening sites in 15 countries in Europe and will recruit patients with all stages of lung cancer and perform molecular profiling analyses to determine whether they would be eligible for inclusion in a targeted therapy clinical trial. Eight patients have already been enrolled and the hope is to recruit 500 patients in the first year then 500–1000 annually thereafter.

## WIN Symposium 2016

The annual Symposium is the most visible part of the WIN Consortium’s activities, but perhaps the most exciting part of WIN is the engagement of all the different parties, from academic institutions to industry collaborators, and the unique opportunity this offers to truly collaborate in developing the next generation of clinical trials. The next WIN Symposium will be held in Paris, 27–28 June 2016 where we will hope to hear of more great progress being made in precision cancer medicine.

## Conflicts of interest

The WIN Symposium 2015 was supported by Illumina, Sanofi, the Fondation ARC pour la Recherche sur le Cancer and educational grants from Amgen, Lily and Novartis. Dr Schilsky is the Chief Medical Officer of the American Society of Oncology and Chairman of the WIN Scientific Advisory Board. He has no financial conflicts of interest.
